# The Use of Fitness Testing to Predict Occupational Performance in Tactical Personnel: A Critical Review

**DOI:** 10.3390/ijerph18147480

**Published:** 2021-07-13

**Authors:** Robin Orr, Takato Sakurai, Jordan Scott, Jason Movshovich, J. Jay Dawes, Robert Lockie, Ben Schram

**Affiliations:** 1 Faculty of Health Science and Medicine, Bond University, Robina, QLD 4229, Australia; takato.sakurai@student.bond.edu.au (T.S.); jordan.scott@student.bond.edu.au (J.S.); jason.movshovich@student.bond.edu.au (J.M.); bschram@bond.edu.au (B.S.); 2 Tactical Research Unit, Bond University, Robina, QLD 4229, Australia; jay.dawes@okstate.edu (J.J.D.); rlockie@fullerton.edu (R.L.); 3 Health and Human Performance Faculty, Oklahoma State University, Stillwater, OK 74074, USA; 4 Department of Kinesiology, California State University, Fullerton, CA 92835, USA

**Keywords:** assessment, task performance, injury risk

## Abstract

Tactical personnel work in an occupation that involves tasks requiring a high level of cardiovascular fitness as well as muscular strength and endurance. The aim of this literature review was to identify and critique studies investigating the relationship between physical fitness, quantified by fitness assessment measures, and occupational task performance. Databases were searched for relevant articles which assessed a fitness measure and a measure of occupational performance. A total of 15 articles were included and were deemed to be of acceptable methodological quality (8.4/12 on the Critical Appraisal Skills Programme checklist). Included articles assessed a variety of fitness attributes and occupational tasks. Across tactical groups, there appear to be no standardized fitness tests that can determine occupational performance, with aerobic fitness, anaerobic fitness, strength, endurance, power, and agility all being associated with occupational task performance. A wide range of fitness assessments appears to be required to predict occupational performance within tactical personnel. Efforts should be made to base fitness assessments on occupational demands unique to both the environment and requirements of each individual tactical unit.

## 1. Introduction

Tactical personnel, including military personnel, law enforcement officers, and firefighters, are required to undergo various physical tasks, all of which involve carrying external loads [1,2]. In military personnel, occupational tasks may include heavy load carriage and mobilizing through difficult terrain while enduring harsh environmental conditions [3,4]. The physical demands of law enforcement duties may include running, restraining perpetrators, self-defense, and manual handling tasks [5,6]. Likewise, firefighters are required to respond to emergency situations requiring search and rescue and protecting community property [7,8]. Firefighters also carry heavy equipment in addition to wearing their own protective gear, while working under severe heat stress at near maximal heart rates for prolonged periods of time [7].

These physically demanding occupational tasks conducted by tactical personnel require a high level of cardiovascular fitness as well as muscular strength and endurance [9,10]. Poor performance in these areas increases injury risk and may lead to mission failure, loss of life, or a perpetrator evading capture [11,12]. For example, Pope et al. [13] and Jones et al. [14] have found that military recruits who had a lower level of cardiovascular fitness, were at increased risk of injury in comparison to the fitter members of their group. Similar results have been found in other fitness measures including power [15], strength [16], and muscle endurance [17]. The relationship between fitness and occupational task performance is highlighted by Robinson and colleagues [18] who found that increased aerobic fitness and strength were associated with better load carriage performance in specialist police. Similarly, the findings from Hendrickson et al. [19] revealed that an 8-week aerobic endurance and strength training led to significant improvements in common tactical occupational tasks including load carriage and repetitive lift and carry tasks.

Due to the importance of fitness on injury risk and occupational task performance, initial trainees seeking employment in tactical populations are required to undergo a series of physical tests. These tests are aimed at assessing future performance [20] and identifying those most at risk of injury [21,22]. Despite the benefit of using many of these assessments, the use of both pushups and situps to determine occupational fitness has attracted wide criticism in the literature [23]. Carstairs et al. [24] found that both pushups and pullups only correlated to one out of four army task simulations. One of the problems identified in these assessments by Blacker et al. [25] was that they are typically performed without any of the additional equipment that tactical personnel are required to carry as part of their occupational requirements.

Although there appears to be a link between different fitness variables and performance in tactical personnel, debate still exists around the assessments used to measure these attributes, and whether a link between these measures of fitness is, in any way, associated with occupationally specific performance tasks. Therefore, the aim of this literature review was to identify and critique studies that investigated the relationships between physical fitness, quantified by fitness assessment measures, and occupational task performance.

## 2. Materials and Methods

### 2.1. Search Strategy

Search terms were developed based on a brief initial review of the literature and in consultation with subject matter experts. Initial terms were adjusted and refined based on the relevance of the re-occurring articles and eventually agreed upon through consensus from all authors. Databases searched included PubMed (https://pubmed.ncbi.nlm.nih.gov/?otool=iaubondlib: accessed on 1 November 2019) EMBASE (https://www-embase-com.ezproxy.bond.edu.au/#/login: accessed on 1 November 2019), and Ebscohost (CINAHL and SportDiscus) (http://web.a.ebscohost.com.ezproxy.bond.edu.au/ehost/search/selectdb?vid=0&sid=d8069b8b-e82d-45c6-96d2-9c8cba027660%40sessionmgr4007: accessed on 1 November 2019). These databases were chosen based on a large number of high-quality peer-reviewed articles present and the representation of journals relevant to the review topic. The finalized search terms and applied filters (where available) for the databases searched are summarised in Table 1.

**Figure 1:**
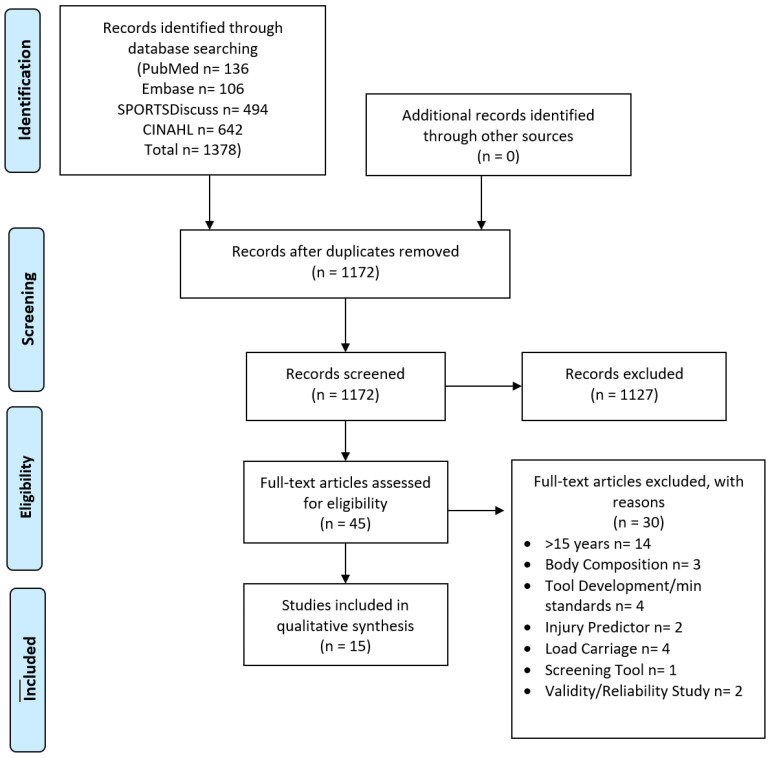
PRISMA [26] diagram summarizing the selection and screening process of the critical review.

After search terms were established and prior to the screening of the studies, inclusion and exclusion criteria (Table 2) were developed. In order to evaluate the most current evidence, studies older than 15 years were excluded during the screening process. Duplicates were removed after the collection of all studies, with the remaining studies screened based on title and abstract for relevance. In order to minimize both search and selection bias, three reviewers were responsible for screening and the selection of relevant studies independently. A search was performed following the Preferred Reporting Items for Systematic Review and Meta-analysis (PRISMA) guidelines. The PRISMA flow diagram [26] (Figure 1) summarizes the entire search process.

### 2.2. Critical Appraisal

All studies which met the criteria were critically appraised using the Critical Appraisal Skills Programme (CASP) checklist for cohort studies [27]. The checklist consists of twelve questions that evaluate the methodological quality of a study. Each question can be answered “yes”, “can’t tell”, or “no”, where one point was given for answers with “yes” and zero-point was given for answers with “can’t tell” or “no”. Questions seven and eight have to be answered with a short response rather than “yes”, “can’t tell”, or “no”; therefore, those two questions were left blank due to subjectivity. Question five and six consisted of two sub-questions “a” and “b” which form a total possible score of 12 out of 12 questions. Methodological quality was also assessed individually by three authors to avoid bias.

### 2.3. Statistical Analysis

Once the critical appraisal score (CAS) for each study was finalized, a mean score for each study was calculated along with a mean and standard deviation of scores for all studies. Krippendorff’s Alpha was used to determine the inter-rater reliability by a fourth author (RO) who was independent of the CASP scoring.

### 2.4. Data Extraction

Following the critical appraisal of all articles, relevant data were extracted under the following headings: Author/population, participants, fitness measure/testing, occupational measures, key results/findings, and average CASP score, and are synthesized in Table 3.

## 3. Results

A total of 1377 studies were identified through the initial search of the four databases. After the removal of duplicates and review by title and abstract, full-text versions for 53 studies were collated for review. These studies were then evaluated against the inclusion and exclusion criteria which left 15 studies remaining for critical review (Table 2). A summary of screening, selection processes, and results of the literature search can be found in the PRISMA flow diagram [26] (Figure 1). Of the 15 studies, seven were on military personnel [24,28,29,30,31,32,33], five on firefighters [34,35,36,37,38], and three law enforcement officers [39,40,41]. Seven studies were from the United States [29,30,35,36,37,39,40], three from Australia [24,33,42], two from UK [33,38], and one each from Finland [31], Sweden [34], and Norway [28]. Seven studies examined male participants [24,28,31,32,33,35,39] while only one study included only female participants [30]. Both males and females were reported on in six of the studies [29,34,36,37,38,40] and one study did not identify the sex of those involved [41].

The mean critical appraisal score (CAS) score for all studies was 8.4 ± 1.2, ranging from the lowest being 6.33 [35] to the highest of 10.0 [29]. The level of agreement between the three raters, as measured by Krippendorff’s Alpha, was 0.80 which was considered to be substantial agreement [42].

### 3.1. Fitness Measures

The most common fitness component measures used were muscular strength assessed in 11 articles [24,29,31,32,33,35,36,37,39,40,41], aerobic capacity, measured in nine articles [29,30,31,33,34,36,37,38,39,40], and muscular endurance, measured in nine articles [24,29,30,31,35,36,37,39,40]. Other measurements of fitness included muscular power which was assessed in six studies [30,32,36,38,40,41] and anaerobic capacity, which was assessed in four studies [29,30,37,38]. The least commonly reported fitness measures were flexibility [29,39] and agility [35,39] both of which were only reported in two studies each.

Muscular strength was measured in various forms across all studies including 1 repetition maximum (1 RM) and 5 repetition maximum (5 RM) measurements, handgrip dynamometry, isometric assessments with chain, and electromechanical dynamometry with isokinetic dynamometry. One-repetition maximum tests were used for exercises, such as bench press [35,39], leg press [37,39], the squat [35], chest press [37], unilateral knee extension [37], and box lifts [24,32]. Other muscular strength measures included handgrip strength [35,36,39,40,41], 5 RM tests for bench press and squat [36], isometric leg and back strength with chain dynamometer [40], and isometric upper and lower body strength with electromechanical dynamometer [31], isometric biceps curl and upright pull [29], squat lift [29], and hip and knee flexor and extensor strength with isokinetic dynamometer [33].

A wide range of aerobic capacity measures was performed including treadmill-based aerobic testing using VO_2max_ [35,38,39], VO_2peak_ [33,39], 3000 m run [31,34], 1.5-mile run [30], 20 m multistage shuttle run and beep test [29,40], the Cooper 12 min run [36], a two-minute arm ergometer assessment at 50 W [29], and a six-minute cycling, six-minute step test, 30 m crawl, and a 500 m rowing test [34].

Muscular endurance was most commonly measured by one-minute pushups, reported in seven articles [24,29,30,31,35,39,40], followed by situps, assessed in six articles [29,30,31,35,39,40], and pullups reported in two articles [31,40]. Other measures of muscular endurance included leg press with 80% 1 RM and chest press with 70% 1 RM [37], maximum repetition of bench press, squat, bent over row, dumbbell biceps curl, and seated dumbbell shoulder press [36].

Power was measured by vertical jump height in three studies [36,40,41], standing long jump in two studies [29,31], and both 2 kg medicine ball put and 9 kg overhead throw in one study [29]. One other article assessed power via a single-leg knee extension power test at 50, 60, and 70% of 1 RM [37].

Anaerobic capacity was measured by either Wingate anaerobic cycling test [28,37], 300 m [28,29], or 400 m sprints [36]. Flexibility was only measured by sit-and-reach in two studies [35,39]. Agility was tested by a change in direction test [39] and Illinois agility test [29].

### 3.2. Occupational Performance Measures

Assessments designed to simulate occupational requirements were used to measure occupational performance in all studies [24,28,29,30,31,32,33,34,35,36,37,38,39,40,41]. Occupational task-specific circuit courses were used in 10 studies [28,30,31,34,35,36,37,38,39,40], while discrete occupational simulation tasks were used in five studies [24,30,33,34,42]. The most common occupational tasks assessment was a simulated victim rescue or drag which was assessed in 11 articles [24,28,29,31,34,35,36,37,38,39,40], followed by a carrying task, assessed in seven articles [30,32,35,36,38,39,41], a loaded stair climb [35,37,38,40] and hose pull and/or drag [35,36,37,38].

Subjective rankings of occupational relevance were assessed in two studies. The evacuation victim drag was subjectively rated as relevant to a ‘large extent’ or ‘very large extent’ by 81% of its participants [28]. The Officer Physical Ability Test (OPAT) for US law enforcement officers was rated as having excellent relevance [39]. Key data pertaining to the fitness measure utilized and the occupational measures conducted are found in Table 3 below.

## 4. Discussion

The aim of this review was to identify and critique studies that investigated the relationships between physical fitness, as measured by fitness assessment measures, and occupational task performance. Overall, the methodological quality of studies in this area appears to be of acceptable quality. Across tactical groups, there appear to be no standardized fitness tests that can determine occupational performance. This finding agrees with previous investigations which have suggested that multi-faceted fitness assessments are important to assess the various essential fitness components of tactical personnel which are often unique to each environment [39].

Aerobic fitness was found to be correlated with OPAT completion time and components of the OPAT [39] and with PAT performance [40] in police officers. It was also related to Military Occupational Specialties test performance [29] and military simulation tests in combat soldiers [31], loaded marches of 3.2 km and 29 km in elite soldiers [33] and field tasks [34], job performance tests [36], PAT [37] and fire fighting simulation tests [38] in firefighters. These results are not surprising given that high levels of aerobic fitness are paramount in tactical professions with research supporting its importance to tasks involving load carriage [18,43]; a common but important requirement within tactical populations. Furthermore, those with lower levels of aerobic fitness must work at a higher level of their overall capacity for a given task, leading to an earlier onset of fatigue [44]. This fatigue may lead to alterations in movement mechanics which in turn leads to injuries. As such, aerobic fitness deficits have also been linked to injury risk in military populations [13,14], Federal agents [45], and firefighters [46]; again highlighting the importance of aerobic fitness for both injury and performance and injury mitigation amongst tactical populations.

In a similar manner to aerobic fitness, measures of strength have also been associated with task performance and injury risk. Load carriage performance and victim drag ability, for example, have both been found to be associated with strength (both relative and absolute) in tactical personnel [18,47]. The carrying of a pack, for example, becomes part of an individual’s body mass, or relative load, hence the relationship with relative strength [48]. Conversely, the victim drag task requires moving of an external or absolute load, hence the relationship with absolute strength. Lower limb muscular strength, specifically, was found to predict dummy drag performance in Navy operators [28], was a predictive component of Military Occupational Specialities tests [29], and correlated to repetitive box lifting tasks in soldiers [32]. Likewise, upper limb strength was correlated with army task simulations [24], ability tests [35], and job performance tests [36] in firefighters. Specific grip strength was associated with improved scores in tactical situations and marksmanship in police officers [41] while finger strength was associated with physical ability tests scores in firefighters [37].

Muscular endurance is often a focus of tactical training programs [49] and bears occupational relevance with the prolonged carrying of stores and pack marching [50]. Upper limb endurance was related to the PAT [40] and components of the OPAT in police officers [39], Army task simulation performance [24] and military simulation tests in soldiers [31], ability tests [35], and job performance tests [36] in firefighters. Likewise, lower limb endurance was related to job performance tests in firefighters [36]. Abdominal endurance was correlated with PAT [40] and OPAT [39] performance while abdominal endurance and strength was found to be important for firefighters’ ability test performance [35]. The use of measures of muscular endurance, such as push-ups or sit-ups, may be more indicative of a global measure of fitness and are, therefore, questioned as relevant in fitness testing [51]. However, this is not to suggest that these measures are not of value, as poor holistic fitness can have second-order impacts on occupational fitness (e.g., increased workplace absenteeism due to illness) [52].

Lower limb power is an occupationally relevant attribute for seeking cover, fire and movement drills, and short sprinting [53,54]. Lower limb power was found to be correlated with evacuation tests in Navy operators [28], PAT performance in police officers [40], and ability tests in firefighters [35]. The ability to generate power in a vertical jump while wearing external load was correlated with military simulation tests [31]. Previous research has identified that declines in power development, measured via vertical jump height, is linked to a significantly greater risk of both injury and the development of illness in police personnel [15] highlighting the benefit of lower limb power as a measure of task performance and injury risk in the tactical field.

Agility was correlated with overall OPAT time and components of the OPAT in police officers [39] while anaerobic power was found to be associated with physical ability test time in firefighters [37] and evacuation tests in Navy operators [28]. No relationship to task performance was found for measures of flexibility in this review, with the flexibility of the hamstrings, in particular, being challenged as a risk factor for injury in general [55], querying the effectiveness of this measure for either injury risk or task performance.

A limitation to this review was the inability to screen for non-English studies which reported on physical fitness and its relationship to task performance. This may have narrowed the body of literature from which conclusions could be drawn. Some further limitations arise from the articles that comprise this review being of only ‘acceptable’ quality. The wide range of fitness assessments studied could be viewed as a limitation. This is most likely due to the wide variety of occupational tasks which occur across the tactical professions, which, while indicative of tactical populations, does make fitness assessment protocol standardization challenging.

## 5. Conclusions

A wide range of fitness assessments appears to be required to predict occupational performance within tactical personnel. Despite aerobic fitness assessments being the most highly studied and closely related to occupational performance, other measures of great importance include muscular strength, endurance and power, agility, and anaerobic capacity. Efforts should be made to base fitness assessments on occupational demands unique to both the environment and requirements of each individual tactical unit.

## Figures and Tables

**Table 1:** Databases and Relevant Search Terms.

Database	Search Terms	Filters	Results
Pubmed	“Police” [Mesh] OR “Military Personnel” [Mesh] OR firefighter OR sheriff OR “incumbent officer” OR “emergency response” AND “Exercise test” [Mesh] OR “Fitness test” [Mesh] AND “Occupation” [Mesh] OR “task performance” OR “work”	Sort by Best Match	136
EMBASE	(‘police’/exp OR ‘military personnel’/exp OR firefighter OR sheriff OR ‘incumbent officer’ OR ‘emergency response’) AND (‘exercise test’/exp OR ‘fitness test’) AND (‘Occupation’/exp OR “task performance” OR work)		106
Ebscohost (both CINAHL and SPORTDiscus)	((“Exercise Test”) OR “Physical Fitness”) OR “Assessment Screen Testing”) AND ((“Police”) OR (“Firefighters”) OR (“Military Personnel”) OR (“Military Recruits”) OR “Sheriff” OR “Incumbent officer” OR “Patrol Officer” OR “law enforcement”) AND (“work * ADJ performance” OR (“Physical Fitness”) OR “occupational ADJ skills” OR (“Task Performance and Analysis”))	Search modes Boolean/Phrase	1136

* Denotes truncation of a word for database searches.

**Table 2:** Inclusion and exclusion criteria and examples.

**Inclusion Criteria**	**Example/s**
Must include a tactical population Must include adult population Must include a physical fitness measure Must include an occupational specific measure Must be full text	Studies including police, military, firefighters Studies including adults (>18 years old) Aerobic fitness, strength, or power MST (Military Simulation Test), PAT (Physical Ability Test)
**Exclusion Criteria**	**Example/s**
Studies older than 15 years Studies used only body composition Studies with tool development Studies with injury predictor Studies used only load carriage Studies used only screening tools Validity and reliability studies	Studies undertaken before 2003 BMI (Body mass index) and fat mass to predict performance Comparing occupational performance measure Analysis of injury risk to performance Load carriage to predict performance FMS (functional movement screen) Studies that looked at validity and reliability of fitness tests or performance measure

**Table 3:** Data extraction table including fitness and occupational performance measures with their key findings.

Author/Population	Fitness Measure	Occupational Measures	Results/Key Findings	Mean CAS
Angeltveit et al. 2016 Norwegian Navy operators	Anaerobic Capacity −30 sec Wingate Test −300 m sprint -Maximum Accumulated Oxygen Deficit (MAOD) test	The Evacuation Test (EVAC) (2 laps of 10 × 20 m W shaped course with a 70 kg dummy (+10 kg plate carrier)	Correlations found between leg strength and power and results of the EVAC test. Wingate test (mean power) r = −0.68, *p* < 0.01 300 m (sprint time) r = 0.51, *p* = 0.04 300 m sprint (mean power) r = −0.67, *p* < 0.01 No correlations with MAOD. Muscle mass, leg strength, and power seem important for determinants of performance in this population.	8.7/12
Beck et al. 2015 USA Male Campus LEO	Flexibility -Sit-and-reach Agility -Change in Direction Agility test Muscular Strength -Absolute and Relative 1 RM bench press -Absolute and Relative 1 RM leg press -Grip Strength Muscular Power -Absolute and Relative Vertical Jump Muscular Endurance -Pushups (maximal reps) -Curlups (maximal reps to cadence) Aerobic Capacity -Graded Treadmill Exercise Test (Absolute and Relative VO_2peak_.	Officer Physical Ability Test (OPAT) Comprised of: -stair ascent (10 stairs) -building entry -stair ascent/descent (14 stairs -barrier jump (0.91 m) -159 m run -multiple barriers (height jump, long jump, crawl, height jump) -victim drag (48.5 kg, 13.7 m) -rescue/arrest -sprint (9.1 m)	Agility and aerobic fitness correlated with total OPAT time. Agility (r = 0.57, *p* < 0.05) Relative VO_2 peak_ (r = −0.65, *p* < 0.05) Agility also correlated with: stair ascent 1 (r = 0.54, *p* < 0.05) stair ascent/descent (r = 0.58, *p* < 0.05) sprint (r = 0.56, *p* < 0.05) Relative VO2 peak correlated with: building entry (r = −0.61, *p* < 0.05) stair ascent/descent (r = 0.67, *p* < 0.01) 159 m run (r = −0.66, *p* < 0.05). Pushups correlated with: building entry (r = 0.62, *p* < 0.05) Curlups correlated to: stair ascent/descent (r = −0.60, *p* < 0.05) 159 m run (r = −0.58, *p* < 0.05) Exercise programs that enhance a variety of fitness characteristics should be used for law enforcement officers.	9.3/12
Carstairs et al. 2016 Male Australian Army soldiers	Task related assessment -Maximal Box Lift and Place Task included lifting a 0.35 × 0.35 × 0.35 m box from the floor to a 1.5 m platform. Weight increased by 5 kg each successful lift. Muscular Strength -Pullups (maximal reps) Muscular Endurance -Pushups (maximal reps in 2 min)	Army Task Simulations: -‘Pack Lift and Place’ (PLP) Progressive lift of a 15 kg pack to a 1.5 m platform, increasing by 5 kg each time to fatigue. -‘Artillery Gunner Loading Simulation (AG)’ Carry a 43 kg ‘shell’ 10 m, place into 1.10 m high tray then perform a 5 kg medicine ball throw. Maximum reps in 10 min -‘Bombing Up an M1 Tank Simulation (M1)’ Carrying a 10 kg ‘shell’ 10 m, then on to a platform 1.70 m high. Progressive increase of 2.5 kg every 10 reps until volitional fatigue. -Bridge Building Simulation (BBS) Carrying a 24 kg bar from the floor 10 m, performing a hang clean then push press. Weight increased by 5 kg each successful lift.	Box lift and place assessment correlated with all simulations PLP (r^2^ = 0.76, *p* < 0.05) AG (r^2^ = 0.36, *p* < 0.05) M1 (r^2^ = 0.47, *p* < 0.05) BBS (r^2^ = 0.63, *p* < 0.05). Pushups correlated with BBS (r^2^ = 0.42, *p* < 0.05) Pullups correlated with BBS (r^2^ = 0.63, *p* < 0.05) Occupational specific assessments show a higher correlation to simulated occupational tasks than generic fitness tests.	8.3/12
Dawes et al. 2017 USA Patrol officers	Aerobic Capacity -20 m Multistage Fitness Test (MSFT) Muscular Strength -Isometric Leg Back Dynamometer -Handgrip Muscular Endurance -Pushups (max reps in 1 min) -Situps (max reps in 1 min) Muscular Power -Vertical Jump	Physical Ability Test (PAT) Tasks included: -unbuckling a seat belt -weaving through cones -stepping through rings -Victim rescue (55 kg) -Carry a crate (18.18 kg) for 6.10 m -barrier jump -ball carry and drop -low crawl -sprint up an elevated ramp -Weighted sled push (~15 m)	PAT performance was best predicted by -MSFT (r = −0.70, *p* < 0.001) -Situps (r = −0.58, *p* < 0.001) -Vertical Jump (r = −0.54, *p* < 0.001) -Pushups (r = −0.52, *p* < 0.001) Aerobic and muscular fitness and anaerobic power are related to occupational performance.	9/12
Foulis et al. 2017 USA Army Combat Soldiers	Muscular Endurance -Pushups (1 min maximal) -Situps (1 min maximal) Muscular Strength -Isometric Biceps curl -Isometric Upright pull -Squat lift (paired dumbbell) -Isometric Handgrip Muscular Power -Powerball throw (9 kg) -Medicine ball put (2 kg) -Standing long jump -Resistance pull speed (45 kg) Anaerobic Capacity -300 m sprint (s): 55.8 ± 7.8 -2 min Arm ergometer (50 W) Aerobic Capacity -Beep test Agility -Illinois agility test	Military Occupational Specialities Tests -Foot march (6.4 km, 43–50 kg of load) -Sandbag carry (carry 16 × 18 kg sandbags 10 m) -Move under fire (small bounds to 100 m, 34–41 kg of load) -Casualty evacuation (progressive move of 23–95 kg through a hole in a platform, 23–95 kg of load) -Casualty drag (drag a123 kg weight 15 m, 34–41 kg of load) -Transfer 30 artillery rounds (30 rounds of 45 kg each) -Stow ammo (move 18 × 25 kg rounds from a rack to a platform over 5 m). -Load main gun (transfer 5 × 25 kg rounds from rack to breach in confined space)	Test Battery 1: Medicine ball put, squat lift, beep test, standing long jump, and arm ergometer. Adjusted R^2^ = 0.80–0.85, *p* < 0.01. Test Battery 2: Medicine ball put, squat lift, beep test, standing long jump. Adjusted R^2^ = 0.79 to 0.80, *p* < 0.01) Test Battery 3: Standing long jump, 1-min push up, 1-min sit up, 300 m sprint, and Illinois agility test. Adjusted R^2^ = 0.55–0.71, *p* < 0.01. Physical training for soldiers should include a combination of strength, power, and aerobic capacity, due to their predictive ability for performance.	10/12
Mitchell et al. 2014 USA Air Force servicewomen	Air Force Physical Fitness Test (AFPFT) Muscular Endurance -Pushups (1 min) -Situps (1 min) Aerobic Capacity -1.5-mile run	Marine Combat Fitness Test (MCFT) -Movement to Contact (MTC) 1/2 mile run -Ammunition Lift (AL) 30-pound weight lifted from chest to above head as many times as possible in 2 min −300 yd Obstacle Course	AFPFT to MCFT r = 0.59 and R^2^ value of 0.35, *p* <.0001. 35% of the variation in MCFT scores could be predicted by AFPFT scores. MTC and AL predicted combat fitness with an adjusted R^2^ of 0.82. Predictability increased using only AFPFT raw scores of the individual events 30lb repetition lift most predictive of combat fitness.	8.7/12
Lindberg et al. 2013 Full-time and part-time Swedish Firefighters	Aerobic Capacity -Submaximal treadmill VO_2max_ -6 min Cycling at 200 W at 60 Revolutions per minute -Crawl 30 m -Run 3000 m -6 min step test (30 steps/min with 24 kg of load) -6 min Treadmill Walking: 4.5 km/h with 24.5 kg of load) -500 m rowing	Firefighting Field Tasks -Cutting (moving an 11 kg concrete saw backward around a 2 × 2 m square 0.05 m above the ground until volitional fatigue) -Stairs (Carry 16 kg basket up 4 floors, 60 secs rest then repeat) -Pulling (Pull a 25 m rope 20 m) -Demolition (16.25 kg bar moved between 1.4 m-1.9 m at 25 lifts/min until exhaustion) -Rescue-(75 kg dummy pulled 30 m) -Vehicle-18.5 kg spreader held against a wall at different points for 15 s until exhaustion -Terrai-(1600 m movement of a weighted basket (18.7 kg) alternating between basket carry and no basket carry)	Both absolute and relative aerobic fitness were significantly correlated with all field tasks. Absolute VO_2max_: cutting r = 0.55, *p* < 0.01 stairs r = −0.75, *p* < 0.01 pulling r = 0.74, *p* < 0.01 demolition r = 0.79, *p* < 0.01 rescue r = 0.79, *p* < 0.01 vehicle r = 0.79, *p* < 0.01 terrain r = −0.79, *p* < 0.01 Relative VO_2max_: cutting r = 0.47, *p* < 0.01 stairs r = −0.52, *p* < 0.01 pulling r = 0.46, *p* < 0.01 demolition r = 0.57, *p* < 0.01 rescue r = 0.57, *p* < 0.01 vehicle r = 0.48, *p* < 0.01 terrain r = −0.74, *p* < 0.01 500 m row time: cutting r = −0.63, *p* < 0.01 stairs r = −0.82, *p* < 0.01 pulling r = 0.76, *p* < 0.01 demolition r = −0.70, *p* < 0.01 rescue r = 0.70, *p* < 0.01 vehicle r = 0.79, *p* < 0.01 terrain r = −0.65, *p* < 0.01 Field tests can predict firefighter occupational performance, with aerobic tests the most valid for predicting occupational performance.	8.3/12
Michaelides et al. 2011 USA Firefighters	Flexibility -Sit-and-reach test Muscular Endurance: -Situps (1 min) -Pushups Muscular Strength -Bench Press (1 RM) -Squat (1 RM) -Isometric Handgrip -Isometric Abdominals Anaerobic Power -Step test (60 sec) -Vertical Jump	Ability Test: -Stair climb -ascend/descend 12 steps × 8 -Rolled hose lift: move 6 rolls of hose (9.53 kg each) from floor to bench to ground -Keiser sled-striking 68.8 kg beam a distance of 1.5 m with a 4.1 kg sledgehammer -Hose pull and Hydrant hook up-Pull fire hose 31.5 m and connect fire hydrant -Rescue Mannequin Drag: Drag 82.5 kg dummy 15.7 m backward -Charged hose advance: lift and carry a hose to water line 15.24 m away	Ability Test completion time associated with -Abdominal Strength (r = −0.53, *p* < 0.01) -Vertical Jump Relative Power (r = −0.44, 0.01) -Pushups (r = −0.27, *p* < 0.05) -Situps (r = −0.41, *p* < 0.01) -1 RM Bench Press (r = −0.41, *p* < 0.01) Abdominal strength, upper body strength, and endurance, and lower limb power are related to improved firefighting performance.	6.3/12
Pihalainen et al. 2018 Male Finnish Soldiers	Aerobic Capacity -3000 m run Muscular Endurance -Pushups (1 min) -Situps (1 min) -Pullups (1 min) Muscular Power -Standing long jump -Counter Movement Jump (loaded and unloaded)	Military Simulation Test (MST): −4 consecutive 6.2 m rushes changing direction after each rush -11.3 m low crawl -sprint 21.8 m -run 21.8 m amd jump over 3 × 40 cm obstacle -lift-carry-lower 2 × 16 kg kettlebells 4 × for 2.5 m -zigzag run of 42.4 m −65 kg dummy drag 24 m in a circle -sprint to start line. Total MST track: 242.5 m	Loaded CMJ, 3000 m run, and pushups were significantly associated with MST time, with muscle mass explained 66% of the variance in MST time. Strongest individual predictor of the MST performance was loaded CMJ (r = −0.66, *p* < 0.001) which explained 47% of the variance in the MST time. Muscle power and endurance capacity are crucial components in anaerobic combat situations.	7.3/12
Savage et al. 2014 Australian Army Soldiers	Muscular Strength 1 RM Test Maximal lifting of a weighted box onto a 1.5 m platform. Dimensions: 0.35 × 0.35 × 0.35 m, metal handles at 0.20 m from base.	Repetitive Box-lift test −6 lifts of between 58–95% 1 RM	Number of repetitions and % 1 RM had strong correlation (r = 0.72, *p* < 0.05) with an adjusted R^2^ of 0.51. no significant difference b/w actual and predicted % 1 RM (*p* > 0.05) 1 RM testing is appropriate for determining physical competency of soldiers.	6.7/12
Orr et al. 2017 Australian Police Recruits	Muscular Strength Isometric Hand Grip	Task Performance Measures -Simulation Task (Basic tactics of defense) -Tactical Options Assessments (TACOPS) (respond to scenarios with an appropriate tactical option) -Marksmanship (scored target shoot with pistol)	Grip Strength related to higher scores in TACOPS -Right Hand (r = 0.227, *p* = 0.003) -Left Hand (r = 0.269, *p* < 0.0001) Grip Strength related to success in TACOPS Right Hand < 30 kg = 44% pass Right Hand > 55 kg = 86% pass rate Grip Strength related to success in Marksmanship Right Hand > 35 kg (r = 0.398, *p* < 0.0001) Left Hand > 35 kg (r = 0.475, *p* < 0.0001) A positive association exists between handgrip strength and police recruit task performance.	9.7/12
Rhea, Alvar, and Gray 2004 USA Firefighters	Aerobic Capacity -Cooper 12 min run Muscular Strength -Bench Press (5 RM) -Back Squat (5 RM) -Isometric Hand Grip Muscular Endurance (to fatigue) -Bench press (45.5 kg) -Back Squat (61.4 kg) -Row (20.5 kg) -Biceps Curl (13.6 kg) -Shoulder Press (11.4 kg) -Handgrip > 25 kg Anaerobic Capacity -400 m sprint	Job Performance Tests: -Hose pull-uncharged fire hose pulled 65.6 m -Stair climb-22 kg hose carried while ascending/descending 5 flights of stairs -Victim drag-80 kg mannequin drag for 30 m while walking backward in full FFs gear -Equipment hoist-Carry 16 kg fire hose up 5 flights of stairs (30.3 m) NOTE: All were performed in turnout clothing with 25 kg tank.	Significant correlations were found between job performance test performance total and Overall fitness (r = −0.62, *p* < 0.05) Bench Press (r = −0.66, *p* < 0.05) Handgrip Strength (r = −0.71, *p* < 0.05) Row Endurance (r = −0.61, *p* < 0.05) Bench Press Endurance (r = −0.73, *p* < 0.05) Bicep Curl Endurance (r = −0.69, *p* < 0.05) Squat Endurance (r = −0.47, *p* < 0.05) 400 m Sprint Time (r = 0.79, *p* < 0.05) Shoulder Press Endurance (r = −0.71, *p* < 0.05) Physical conditioning programs for firefighters should address all components of fitness.	9.7/12
Sheaff et al. 2010 USA Firefighters	Muscular Strength -Chest Press (1 RM) -Leg Press (1 RM) -Unilateral knee extension -Grip Strength Muscle Endurance -Chest Press (70–80% 1 RM) -Leg Press (70–80% 1 RM) Muscle Power Knee extension (50–70% 1 RM) Anaerobic Capacity -Wingate Anaerobic Test Aerobic Capacity -Graded treadmill exercise test -Stair climb via a Stairmaster	Candidate Physical Ability Test (CPAT) -8 firefighting tasks while wearing a 22.7 kg load Stair climb (60 steps/min for 3 min) with 11.3 kg weight vest Hose drag (61 m hose dragged 45.7 m with turns) Equipment carry (carry 2 saws 150 ft) Ladder raise and extension (7.5 m ladder) Forcible entry (Hitting wall with a sledgehammer) Search (crawl through 19.5 m tunnel maze) Rescue (drag 61.2 kg mannequin 21.4 m) Ceiling breach and pull (raise a door multiple times)	Anaerobic Power, aerobic power and strength all associated with quicker CPAT times Wingate mean power (r = −0.664, *p* < 0.001) 1 RM Chest Press (r = −0.485, *p* < 0.001) Absolute VO_2max_ (r = −0.602, *p* < 0.001) Isometric Finger Strength (r = −0.500, *p* = 0.009) Best predictors of CPAT performance = Absolute VO_2max_ and anaerobic fatigue resistance during Wingate (Adjusted R^2^ = 0.817, *p* < 0.001). Anaerobic and aerobic fitness best predict overall CPAT performance.	9.3/12
Siddall et al. 2018 UK Firefighters	Aerobic Capacity Graded Treadmill Exercise Test	Fire Fighting Simulation Test (FFST) 1-Equipment carry: 25 kg over 200 m 2-Casualty evacuation: 75 m hose drag, 25 m unladen then 55 kg dummy drag 50. 3-Hose run: Simulation, 100 m water relay (4 × 25 m hose ~13 kg). Consists of 8 × 25 m unladen traversals (200 m) at both the start and end, four 25 m traversals (100 m) carrying two hoses, two 25 m traversals (50 m) carrying one hose, two 25 m unladen traversals (50 m) and four 25 m traversals (100 m) rolling out hose, totaling 700 m.	Relative VO_2max_ (r = −0.711) had a stronger inverse relationship with FFST completion time than absolute VO2max (r = −0.577) explaining ~18% more of the variance in FFST performance. Fitter individuals were able to complete the Firefighter Simulation Test more quickly.	8/12
Simpson, Gray and Florida-James 2006 Male elite units of the British Army	Muscular Strength Concentric hip and knee flexors and extensors via isokinetic dynamometry Aerobic Capacity Treadmill Graded Exercise Test	Backpack run test 2-mile (3.2 km) run with 20 kg backpack. Time Trial 29 km time-trial over hills with speed marches over prominent peaks with 20 kg backpack.	Isokinetic strength did not correlate with any of the tests. Test duration on treadmill test correlated with 2-mile backpack run (r = −0.57) and 29 km time trial (r = −0.66). Absolute (r = −0.06) and relative VO_2peak_ (r = −0.08) were poorly associated with 2-mile backpack run test and time trial (r = −0.12 & r = −0.37 respectively). The maximal treadmill test and 2-mile backpack run are useful indicators of performance in an arduous hill march.	7/12

CAS = Critical Appraisal Score: RM = Repetition Maximum.

## Data Availability

Data sharing not applicable.
